# The History of Carbohydrates in Type I Allergy

**DOI:** 10.3389/fimmu.2020.586924

**Published:** 2020-10-09

**Authors:** Miriam Hils, Florian Wölbing, Christiane Hilger, Jörg Fischer, Nils Hoffard, Tilo Biedermann

**Affiliations:** ^1^Department of Dermatology and Allergy Biederstein, School of Medicine, Technical University of Munich, Munich, Germany; ^2^Department of Infection and Immunity, Luxembourg Institute of Health (LIH), Esch-sur-Alzette, Luxembourg; ^3^Department of Dermatology, Faculty of Medicine, Eberhard Karls University Tübingen, Tübingen, Germany; ^4^Clinical Unit Allergology, Helmholtz Zentrum München, German Research Center for Environmental 10 Health GmbH, Neuherberg, Germany

**Keywords:** alpha-gal, carbohydrate, allergen, crossreactive carbohydrate determinants, glycolipid, glycoprotein, IgE, type I allergy

## Abstract

Although first described decades ago, the relevance of carbohydrate specific antibodies as mediators of type I allergy had not been recognized until recently. Previously, allergen specific IgE antibodies binding to carbohydrate epitopes were considered to demonstrate a clinically irrelevant cross-reactivity. However, this changed following the discovery of type I allergies specifically mediated by oligosaccharide structures. Especially the emerging understanding of red meat allergy characterized by IgE directed to the oligosaccharide alpha-gal showed that carbohydrate-mediated reactions can result in life threatening systemic anaphylaxis which in contrast to former assumptions proves a high clinical relevance of some carbohydrate allergens. Within the scope of this review article, we illustrate the historical development of carbohydrate-allergen-research, reaching from only diagnostically relevant crossreactive-carbohydrate-determinants to clinically important antigens mediating type I allergy. Focusing on clinical and immunological features of the alpha-gal syndrome, we highlight the discovery of oligosaccharides as potentially highly immunogenic antigens and mediators of type I allergy, report what is known about the route of sensitization and the immunological mechanisms involved in sensitization and elicitation phase of allergic responses as well as currently available diagnostic and therapeutic tools. Finally, we briefly report on carbohydrates being involved in type I allergies different from alpha-gal.

## Introduction

A key function of the immune system is to distinguish self from altered-self and non-self in order to subsequently induce tolerance or a specific immune response, respectively. Environmental factors like pollen or food are potential allergic substances which are normally tolerated by the immune system. However, in some individuals, the immune system mounts a type-2 biased reaction in response to such factors. Type 2 immune responses comprise, among others, Th2 cells, type 2 innate lymphoid cells (ILC2) and basophils. As hallmark type 2 cytokine, IL-4 drives the switch in B cells to the production of allergen-specific IgE antibodies which are bound by the high affinity FcεRI on mast cells and basophils allowing the elicitation of immediate allergic reactions that are called type I in contrast to e.g., directly cell-mediated type IV allergic reactions. Subsequent exposure to the allergen results in cross-linking of these FcεRI-bound IgE followed by the release of vasoactive substances such as histamine by the mast cells and basophils which in turn mediate typical type I allergy-associated local and systemic symptoms, at worst, anaphylaxis. While, in the past, mainly proteins have been described as allergy-eliciting components within pollen, venom or food, carbohydrates have been considered as non-immunogenic and thus negligible in the promotion of allergic responses. However, recent observations clearly show that carbohydrates as well as glycolipids are involved in sensitisation as well as elicitation of hypersensitivity reactions. Especially the identification of alpha-gal as the epitope responsible for triggering anaphylaxis in response to red meat, innards and therapeutical monoclonal antibodies such as cetuximab, drastically changed the accepted view of carbohydrates as allergens [for an overview see also (1–4)]. How the role of glycans as allergens changed over time and the immunological mechanisms involved in sensitisation to as well as elicitation of allergic responses by carbohydrate allergens will be discussed in this review.

## Carbohydrate Function and Structure

**Figure 1:**
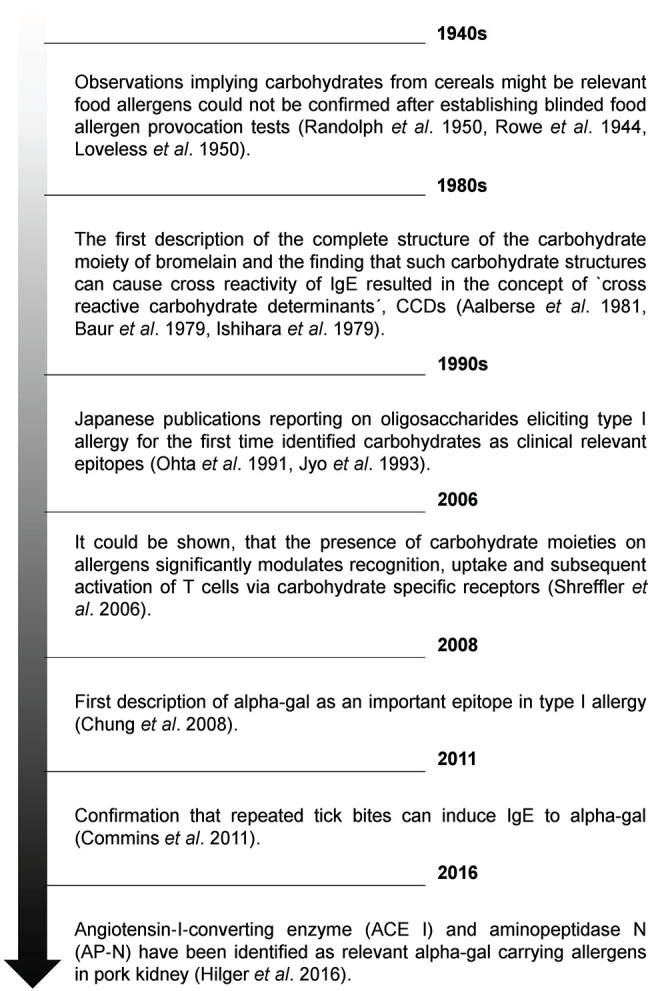
Timeline showing relevant milestones in understanding the role of carbohydrates in allergology.

Carbohydrates are organic biomolecules consisting of one or more simple sugars. These so-called monosaccharides like glucose, fructose, mannose or galactose are built according to the very basic formula C_n_H_2n_O_n._ By N-, C-, or O-glycosidic linkage, monosaccharides can be coupled to form disaccharides, oligo- and polysaccharides or complex biomolecules with non-sugar constituents (5). At a first glance the most important function of carbohydrates seems to be the storage of energy and assembly of structural components like cellulose in the cell walls of plants (6). However, as independent molecules but even more as side chains of peptides, proteins or lipids, so called glycopeptides, glycoproteins and glycolipids, carbohydrates have crucial functions in e.g., development, immune regulation, blood clotting and many other vital physiologic processes. Accordingly, changes in glycosylation patterns have severe and systemic consequences resulting in disease (7). Peptides and proteins exhibit so-called glycosylation sites provided by, if accessible in the final tertiary structure, certain amino acids. In principle, carbohydrates can be bound N-linked to a nitrogen of the amino acids arginine or asparagine, O-linked to a hydroxyl group of tyrosine, serine, threonine or hydroxyproline or, much less common, C-linked to a carbon of tryptophan (8). Therefore, most larger proteins potentially qualifying as allergens possess one or more glycosylations. In addition, it is well-known that especially carbohydrate determinants are potent immunogens with a broad clinical relevance e.g., as vaccination antigens like bacterial polysaccharides being part of the Haemophilus influenza type b vaccine or as blood group antigens (9). Strikingly, it could also be shown that especially carbohydrate antigens might serve as triggers of Th2 immunity (10, 11). Consequently, a glycan-related IgE-reactivity has been demonstrated in most allergen sources (12). Likewise, carbohydrates and their possible role in allergy became an early focus of allergy research (for an overview, see Figure 1).

## The Historic View of Carbohydrate Allergens

In the 1940's a series of articles reported an important role of corn starch and sugars from cereals as food allergens (13, 14). Because of hearings at the US Food and Drug Administration in 1949, where the advisability of labeling foods was discussed from the perspective of allergy research, these publications received a lot of attention. However, some of these reports referred to quite unspecific symptoms for diagnosing allergy to starches, syrups and sugar from cereals and could not be confirmed by most contemporary specialists. Interestingly, the controversy about the relevance of the reported observations resulted in the development of the most objective diagnostic procedure for food allergy, the blinded provocation test. This even led to the appeal to introduce such controlled, objective methods to study food allergy. Using the new established method, by the way, an important role of corn starches or sugars as allergens could not be confirmed (15). At the same time Coulson et al. reported evidence that carbohydrates associated with allergenic proteins (they investigated cottonseed allergens) do a) not determine the antigenic specificity and b) not influence the “shocking capacity” of the allergenic protein (16). Both findings together indicated that carbohydrates do not play a major role as allergens.

## Cross Reactive Carbohydrate Determinants

**Figure 2:**
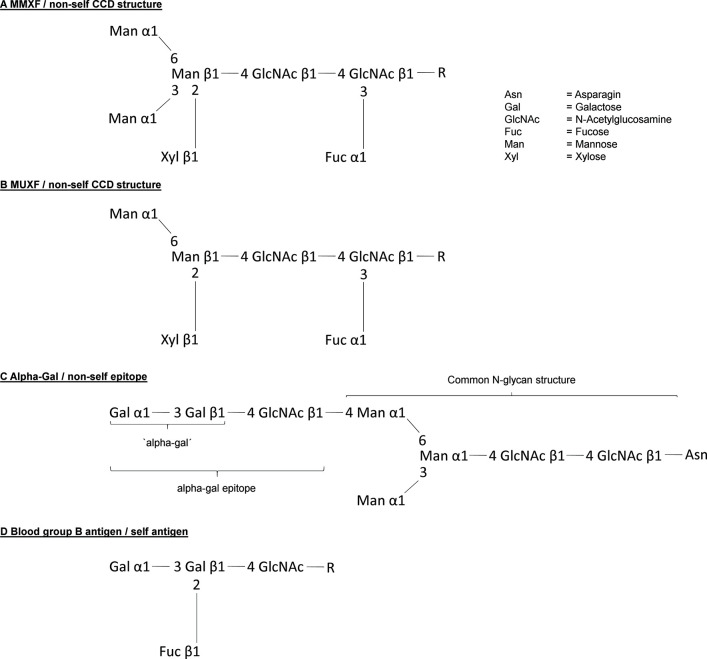
Structure of the most relevant carbohydrate allergenic structures. **(A,B)** Structure of the CCDs MMXF and MUXF. **(C)** Structure of the allergen alpha-gal and **(D)** shows in comparison the difference to the blood group B antigen.

In the 1960's, scientists increasingly hypothesized that allergens must be characterized by a common feature defining an allergen as an allergen (17). Several groups systematically investigated the clinical reactivity of allergic patients to different allergen extracts in cutaneous tests and, according to the calculated correlation coefficients, proposed that allergens can be grouped into clusters or families of closely related allergenic potential (18). Based on these results, others hypothesized that the crucial chemical properties of allergens are N-glycosidically linked sugars (19). Although this did not turn out to be the key to understand the allergic potential of certain antigens in general, it proved to be correct: Many allergens, especially those from the plant kingdom, possess common N-glycosidically linked immunogenic carbohydrate determinants with IgE binding properties. In 1979, a Japanese group described the complete structure of the carbohydrate moiety of stem bromelain, which is generally seen as the starting point of identifying cross reactive carbohydrate determinants and their relevance (20). In the same year, Baur et al. described a mutual inhibition of Radio-Allergo-Sorbent-Test (RAST) to papain, bromelain, wheat flour, rye flour, grass pollen, and birch pollen (21). However, the relevant cross-reactive structures were difficult to identify with radioimmunoelectrophoresis, which was the standard method at the time. In the early 1980's, Aalberse and his colleagues identified IgE antibodies that crossreact with vegetable foods, pollen, and Hymenoptera venom with the new and preferred procedure “immunoblotting” (22). Especially two findings allowed to establish the concept of ‘cross reactive carbohydrate determinants’, shortly CCDs: Their results showing that the cross-reactive inhibitory effect of grass pollen in RAST could be destroyed by periodate pretreatment, a procedure resulting in breakdown of carbohydrates, and observations, that lectin containing gums like tragacanth gum - lectins very specifically bind certain carbohydrates - can inhibit RAST levels to buckwheat or potato. The findings of Aalberse et al. were supported by following publications showing that IgE from patients allergic to honeybee venom binding to phospholipase A 2 (PLA 2; Api m 1) reacts with the same CCD which is also present on plant glycoproteins (23, 24). Later, these findings were proven by showing that glycopeptides made from pineapple stem Bromelain can inhibit IgE binding to Api m 1. Immunogenicity of CCDs is caused by differences in the cascade of synthetisation. The initial steps of protein N-glycosylation are essentially conserved in all eukaryotic organisms (9, 25), however, the following steps differ between ‘higher animals’, i.e., the deuterostomia on the one hand and the protostomia (e.g., insects), the acoelomata (e.g., many parasitic worms) and the plants on the other hand (9). Therefore, the prototypic N-glycan CCD structures MMXF 3 and MUXF 3 (Figures 2A,B) exhibited by horse radish peroxidase (HRP) or bromelain, respectively, show non-human linkage: Fucose alpha 1,3 is linked to the core region of glycoprotein N-glycans. In humans, the N-glycan core fucose is linked to position 6 of the first GlcNAc unit. The non-human monosaccharide β-(1,2) xylose is bound to the first mannose in the N-glycan core region (26, 27). These immunogenic fucose and xylose moieties have been identified responsible for IgE binding and IgE cross-reactivity. It is expected that about 15–30% of atopic patients mounting IgE responses have anti-CCD IgE (24, 28–30). In principle, binding to anti-CCD IgE is able to cross-link FceRI and activate human mast cells and basophils (29, 31, 32). However, in comparison to protein antigens usually much higher concentrations, up to 10-fold, are necessary demonstrating low potency (31, 33, 34). Thus, there is broad consensus that anti-CCD IgE generally does not cause clinical symptoms when crosslinked by CCDs even though single cases remain, in which a clinical relevant anaphylactic potential is still debated. Interestingly, recent publications also discuss the possibility that CCDs might have a clinical relevance by rather being disease protective (35, 36). Nkurunungi et al. could show, that in ugandan schoolchildren the presence of IgE to a subset of core α-1,3-fucose substituted N-glycans was lower in the asthmatic population. However, those are initial observations which at first should be corroborated by more mechanistic studies.

## Carbohydrates as Important Epitopes in Type I Allergy

**Figure 3:**
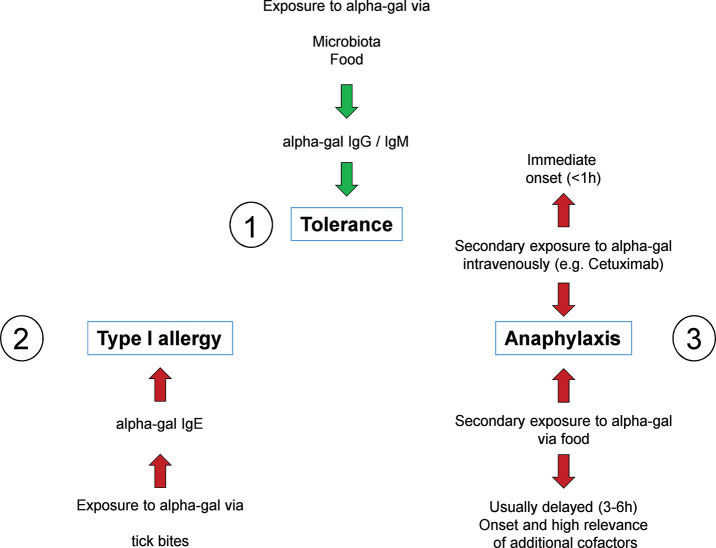
The alpha-gal syndrome. All individuals initially develop tolerance to alpha-gal mediated by constant exposure through bacterial colonization of the intestine and potentially also to food, associated with alpha-gal specific IgG and IgM antibodies (1). However, in some individuals, repetitive tick bites can result in a break of tolerance by induction of alpha-gal specific IgE (2). Upon consumption of red meat and innards as well as administration of alpha-gal containing drugs such as the therapeutic monoclonal antibody cetuximab, these individuals experience symptoms up to fatal anaphylaxis. While symptoms in response to cetuximab occur immediately after administration, anaphylaxis in response to red meat or innards occurs in a delayed fashion 3–6 h after consumption (3).

Understanding the role of IgE recognizing CCDs and its structures allowed to crucially improve allergy diagnostics. However, it might have delayed the understanding of a possible role of carbohydrates as clinically relevant allergens (9). This has changed drastically since in 2009 Commins et al. reported that the carbohydrate allergen galactose-α-1,3-galactose (Figure 2C) can induce severe anaphylaxis. Specifically, IgE antibodies to the carbohydrate galactose-α-1,3-galactose (alpha-gal) were found to elicit serious, even fatal, reactions to the monoclonal antibody cetuximab (37). Alpha-gal is a carbohydrate broadly expressed, even by bacteria, and can be found in high density in red meat and especially in innards like kidney and patients with alpha-gal allergy were increasingly recognized (38–41). However, alpha-gal has been recognized as a relevant antigen much earlier. By investigating the cross reactivity between human sera and animal blood, Landsteiner and Miller (42) discovered that ‘in twelve species of seven genera of Platyrrhina (New World monkeys) […] a factor similar to the human isoagglutinogen B was present; in 18n species of four genera of Cercopithecidae (Old World monkeys) it was absent, although the latter are more closely related to man than the former’ (42). Indeed, the alpha-gal epitope is highly similar to the human blood group B antigen (Figure 2D). Roughly at the same time as the role of carbohydrate antigens as CCDs in 1983. Galili et al. observed a high number of IgG antibodies directed to alpha-gal, initially in thalassemia patients, representing up to 1% of all IgG (43). Further investigations showed that these antibodies very specifically recognize only the alpha-gal epitope showing no cross reactivity with most similar carbohydrate epitopes (44, 45). The development of anti-alpha-gal antibodies is the result of an evolutionary loss of alpha-gal epitopes due to frameshift mutations in the α-1,3-galactosyltransferase gene in humans and old world apes between 25 and 40 million years ago (46). As a consequence, alpha-gal is recognized by the human immune system as a non-self and potentially harmful molecule capable of triggering an immune reaction. It is not totally understood why this mutation could prevail, but it must have provided an evolutionary survival advantage. Indeed, anti-alpha-gal IgG antibodies significantly improve the immune defense against pathogens expressing the epitope like *Plasmodium species* and *Trypanosoma cruzi* (47–49). Accordingly, human anti-alpha-gal IgG were also shown to bind to a number of Gram-positive and Gram-negative bacterial pathogens (41). After its discovery, potential clinical implications of the alpha-gal epitope remained elusive for many years. While a role in immune reaction to cancer cells has been suspected early and is still discussed, alpha-gal initially became most prominent as a relevant xenoantigen impairing efforts to establish xenotransplanation from pig to human (50). Thanks to the extensive research in this field, precious knowledge about the biology of alpha-gal, especially organ and species specific expression patterns and specificity of antibodies, had been gathered even before the discovery of alpha-gal allergy. Xenotransplantation research also led to the generation of α-1,3-galactosyltransferase and consequently also alpha-gal deficient mice and pigs (51–53). It was a surprise when in 2008, Chung et al. observed that in some patients who developed a severe anaphylaxis following even the very first administration of the new epidermal growth factor receptor (EGFR)-antibody cetuximab, the relevant epitope turned out to be alpha-gal and that affected patients seemed to have preformed IgE directed to it (37). Further research, inspired by the observation that ‘the geographical distribution of cases matched the reported distribution of a tick-borne disease called Rocky Mountain spotted fever’ which is transmitted by bites of *Amblyomma americanum* (the lone star tick), revealed that indeed tick bites seem to have the potential to induce IgE production to alpha-gal. In 2011, Commins et al. could show that the serum concentration of IgE directed to alpha-gal and IgE reacting with extract of the lone star tick significantly correlate (54). In addition, it could be shown that alpha-gal is present in the gut and in the salivary glands of the ticks *Amblyomma americanum* and *Ixodes ricinus*, the latter being endemic in Europe (55–58). Finally, repeated tick bites can booster the immune reaction to alpha-gal and vice versa subjects living in areas void of ticks do not develop IgE to alpha-gal. Because allergy to alpha-gal means not just red meat allergy but also allergy to other alpha-gal residue carrying substances like gelatin or recombinant pharmaceuticals from mammalian cells and sometimes also comprises allergic reactions to tick bites, it is also called “alpha-gal syndrome” (59, 60) (summarized in Figure 3). Next to the induction by repeated tick bites, clinical hallmarks are a typically relative high age of about 50–60 years confirmed in most cohorts and an often delayed onset following meat consumption of at least 2 h. Interestingly, after consumption of pork kidney allergic reactions can occur within < 1 h (61–63). These differences in latency might be caused by the required digestion, absorption and conversion of alpha-gal containing glycoproteins and / or -lipids crucially preceding their recognition by FcεRI-bound IgE on mast cells and basophils. While most ingested proteins are transported to the bloodstream within 1–2 h after ingestion, the majority of lipids enters the blood as part of chylomicrons around 4 h postmeal (64). Indeed, using proteins and lipids extracted from grilled beef, Roman-Carrasco et al. demonstrated that alpha-gal containing glycolipids but not -proteins were able to cross a monolayer of intestinal cells as part of chylomicrons (65). As relevant alpha-gal carrying allergens in pork kidney, angiotensin-I-converting enzyme (ACE I) and aminopeptidase N (AP-N) have been identified (66). The clinical diagnosis is based on a case history with occurrence of systemic symptoms of type I allergy after consumption of red meat or offal and the presence of specific IgE to alpha-gal. The most commonly used commercially available reagent for detecting alpha-gal specific IgE is native bovine thyroglobulin. Prick test solutions authorized and available to confirm meat allergy show a low sensitivity in alpha-gal allergic patients and are usually not suitable to prove allergy to alpha-gal (61). In contrast, prick-to-prick tests, especially those using fresh pork or beef kidney lysate or red meat, as well as intradermal test with Cetuximab or 4 % gelatin polysuccinate have shown to provide a higher sensitivity and can be helpful to diagnose alpha-gal syndrome (61). Importantly, a relevant portion of individuals with IgE to alpha-gal remains clinically tolerant with no symptoms of alpha-gal syndrome upon exposure. In a cohort of German forest workers, the prevalence of IgE to alpha-gal was 35% but only 8.6% of the participants with alpha-gal-sIgE levels ≥0.35 kU_A_ /L had a manifest allergy to alpha-gal (67). Therefore, as established in the early 1950's, oral provocation tests are the tool to establish the clinical relevance of IgE sensitization to alpha-gal. As elicitation of clinical symptoms depends on the presence of additional augmentation or cofactors like exercise, alcohol consumption or intake of non-steroidal anti-inflammatory drugs in some alpha-gal allergic patients, titrated exposure to such cofactors should be included in the test procedure (61, 68, 69). Interestingly, it might be possible to distinguish patients with a relevant sensitization from those with IgE to alpha-gal but persistent tolerance by performing basophil activation test (BAT). Mehlich et al. reported that alpha-gal allergic patients showed a significantly higher%CD63/anti-FcεRI ratio using either alpha-gal-HSA, pork kidney extract or bovine thyroglobulin than alpha-gal tolerant patients also having IgE to alpha-gal (70).

Regarding basic research, data on allergy to alpha-gal in animal models allowing a better understanding of underlying immune mechanisms is still sparse. However, as a proof of concept Araujo et al. could show that effective sensitization of α-1,3-galactosyltransferase deficient mice to alpha-gal can be achieved by either subcutaneous injection of alpha-gal carrying virus like particles, each displaying 540 copies of alpha-gal on its surface or by injection of tick saliva or by feeding ticks on the back of a mouse for 9 days using a feeding chamber. An Elisa specific for anti-alpha-gal antibodies showed a strong induction of alpha-gal specific IgE in mouse serum after tick feeding but a clearly less effective induction following subcutaneous sensitization using virus like particles or tick saliva (71). Our own unpublished results confirm that a percutaneous sensitization to alpha-gal in mice is effective and induces clinically relevant allergy in mice, however, further research is necessary to understand how allergy to alpha-gal is induced and how it might be prevented or more efficiently treated.

Interestingly, clinically relevant type I allergy to carbohydrates other than alpha-gal has also been reported but this is much less well-understood than the alpha-gal syndrome (72). Initially, in the 1980's in workers on Japanese oyster farms, a noticeable increase of occupational asthma has been observed. Analyzing these cases, Ohta et al. could identify a number of different oligosaccharides isolated from the H-antigen of the sea squirt as the relevant allergens (73, 74). Some of these oyster farm workers later on also suffered from anaphylactic reactions after drinking a lactic acid beverage especially popular in Japan. In these patients 1–3 or 1–6 linked so called galacto-oligosaccharides (GOS) consisting of four saccharides elicited positive results if used in skin-scratch tests and in a histamine release assay. GOS consist of 2-6 mostly galactose molecules and a terminal glucose but can significantly vary in length and type of linkage between the monomers (75). Most interestingly, IgE antibodies directed to these GOS also cross-react to the sea squirt antigens (76). In addition, allergic reactions to other GOS used as probiotic supplements in beverages, infant milk products or commercially available milk drinks even outside of Japan have been reported (77, 78). Taken together, these observations demonstrate that other type I allergies to carbohydrates than that to alpha-gal exist. Indeed, it seems likely that the increasing understanding about the immune mechanisms underlying allergy to carbohydrates results in identification of additional and clinically relevant carbohydrate allergens.

## Today's View of Carbohydrate Allergens

**Figure 4:**
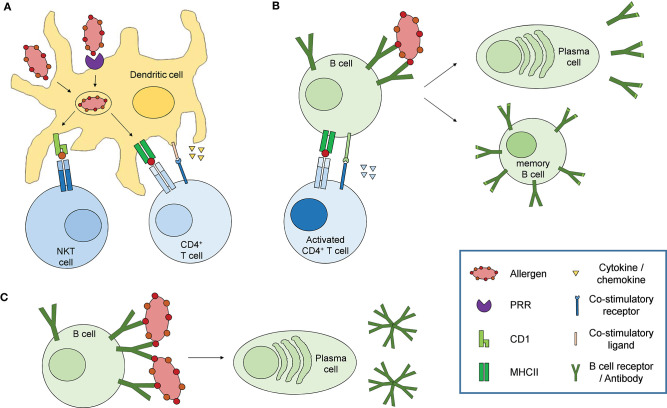
Immune response to carbohydrate antigens. **(A)** Dendritic cells sense carbohydrate constituents on allergens via pathogen recognition receptors (PRR) such as C-type lectin receptors, leading to their activation. Allergens are taken up by receptor mediated endocytosis or phagocytosis, processed and presented on MHCII (glycoproteins, defined polysaccharides) or CD1 (glycolipids) to specifically activate CD4^+^ T cells or NKT cells, respectively. Activated dendritic cells additionally secrete different cytokines and chemokines and express co-stimulatory molecules which initiate and modulate the adaptive immune response. **(B)** Activated CD4^+^ T cells in turn activate naïve B cells recognizing and presenting the same allergen on MHCII via co-stimulatory signals and secretion of specific cytokines resulting in affinity maturation, class switch recombination, memory B cell and plasma cell differentiation and antibody secretion. **(C)** Specific B cell subsets, mainly B1 cells, can be activated in a T cell independent fashion via extensive cross-linking of the B cell receptors by repetitive epitopes, resulting in the secretion of so-called natural antibodies characterized by only low antigen affinity and mainly IgM isotype.

Immune responses associated with allergy are in most cases elicited by binding of the allergenic substance to specific IgE antibodies coupled to the high affinity IgE receptor FcεRI on mast cells and basophils, leading to the release of mediators such as histamine. Thus, an induction of a type 2-dominated immune response and the associated polarization of CD4+ T cells to become Th2 cells and the production of type 2 cytokines, among them IL-4, can be anticipated to be underlying the class switching of allergen-specific B cells to produce IgE antibodies. To allow the Th2 cell development, IL-4 as dominant education factor is crucial. One possible initial cellular source of IL-4 are the basophils which are well-known for their substantial role in allergic inflammation as well as in parasitic infections (79–82). Basophils have even been described to function as non-professional antigen presenting cells able to take up, process and present allergen on MHCII and induce Th2 responses in mouse models of papain immunization and ovalbumin-induced food allergy, however, their role in “real life” Th2 responses is still a matter of debate (83, 84). Of note, most allergens are proteins, however, the majority of allergens from sources such as pollen, food and insect venom also carry carbohydrates and (glyco-)lipids. While the cascade of events leading to adaptive immune responses to protein antigens are increasingly well-understood (85), the mechanisms underlying carbohydrate-specific humoral and cellular immune reactions are less well-defined (summarized in Figure 4). Innate immune cells recognize so-called pathogen associated molecular patterns (PAMPs) on pathogens leading to their activation and subsequent initiation of the adaptive immune response. Pathogens are taken-up and processed by activated antigen presenting cells (APCs), of which the dendritic cells are the most specialized, which in turn present pathogen-derived peptides on MHCII molecules. Dendritic cells subsequently migrate to draining lymph nodes where they initiate the adaptive immune response by activating antigen-specific naïve T cells which specifically recognize the presented peptide-MHCII complex. T cells in turn differentiate into specialized effector T cell subtypes, depending on the cytokine milieu in the cellular environment. Antigen-specific B cells in the lymph node are subsequently activated by a specialized subset of T helper cells, the follicular helper cells, which provide both cytokines and co-stimulatory molecules leading to B cell activation, proliferation, plasma cell differentiation, antibody secretion and memory formation (85).

## Carbohydrate Specific Antibody Responses – Requirement for T Cells?

While efficient antibody responses to protein antigens in general require T cell help and thus presentation of antigenic peptides via MHCII, B cell activation mediated by carbohydrate antigens, according to traditional views, has been suggested to occur independent of T cells due to extensive cross-linking of B cell receptors by repetitive glycan epitopes (Figure 4C) (85, 86). Consequently, antibody responses to carbohydrate antigens differ strongly from those to protein antigens. First, carbohydrate-specific antibodies use a restricted panel of variable gene pairs as well as confined antibody isotypes, with an overrepresentation of IgM and defined IgG isotypes, namely IgG2 (human) and IgG3 (mouse) (87–89). Due to their capability to form antibody multimers, the overrepresentation of IgM and IgG2 or IgG3 isotypes provides carbohydrate-specific antibodies with the capability to efficiently bind to multivalent glycan structures on the surface of microbes, cells and glycoproteins. Second, mainly due to the seemingly limited contribution of carbohydrate antigens to memory responses and the resulting limited affinity maturation, carbohydrate-specific antibodies primarily exhibit only low affinity compared to antibodies recognizing protein antigens. These characteristics are the main reason for the usage of thymus-dependent forms of vaccines to capsular bacteria such as *Haemophilus influenzae* type b, where carbohydrate-protein conjugates are used to facilitate higher antibody affinities as well as memory responses (90, 91). However, more recent studies described a similar affinity of carbohydrate-specific antibodies of IgE and IgG isotypes for the CCDs α1,3-fucose and xylose as well as high affinity binding of anti-alpha-gal and anti-LPS antibodies (92–94). T cell-independent natural antibodies are primarily produced by B1 cells in response to damage-associated molecular patterns (DAMPs). Interestingly, glycoproteins and glycolipids in vertebrates primarily terminate with sialylic acid residues, which are recognized as normal self by various receptors such as Siglecs (95) while asialylated glycoproteins e.g., with terminal galactose are recognized as altered self or DAMPs. Indeed, anti-alpha-gal IgG and IgM are highly abundant antibodies, originally sorted into the pool of natural antibodies, and are present in all humans with up to 1% of B lymphocytes specific for alpha-gal (44, 96). Of note, it has been described that B1 cells can enter germinal center reactions under certain circumstances, particularly in autoimmune conditions like systemic lupus erythematosus (SLE), allowing class switch recombination and somatic hypermutation, leading to high affinity IgG or IgA responses (97). Thus, B1 cells are likely involved in antibody responses to carbohydrate allergens, especially in those to alpha-gal. In contrast to these observations, using Ggta1 Tcrβ double deficient mice, Cretin et al. showed that antibody responses to alpha-gal are depending on T cells. They observed an increase in alpha-gal -specific IgM titers with age in T cell carrying but not T cell deficient Ggta1 ko mice. Additionally, immunization with pig cells boosted the alpha-gal-specific antibody response only in mice carrying T cells and blocking CD40-dependend co-stimulation abolished the increase in IgM titers in Ggta1 ko mice, indicating that antibody responses to alpha-gal require T cell help (98). Indeed, some carbohydrate antigens, especially zwitterionic polysaccharides from the capsules of some bacteria, have been shown to be presented by MHCII molecules to activate CD4 T cells (99–101), indicating that at least some carbohydrates can be presented by MHCII molecules in certain conditions. Cobb et al. showed that polysaccharide A from *B. fragilis*, a zwitterionic polysaccharide, can be taken up, processed and presented on MHCII by human as well as murine B cells. Confocal microscopy of splenocytes from PS-A treated mice confirmed the formation of an immunological synapse between polysaccharide-MHCII complexes on professional antigen-presenting cells (APCs) and the corresponding T cell receptors (αβTCRs). Specific glycoproteins and glycoconjugates can also be presented via the classical MHCII pathway and in turn activate specific T cells, whereby the glycan group can either be preserved, converted or removed from the presented peptide fragment (102–105). Most interestingly, carbohydrate-dependent proliferation of T cells from bee venom allergic patients in response to the allergen phospholipase A2 derived of bee venom has been demonstrated (102). Thus, carbohydrates with certain molecular characteristics as well as glycopeptides can indeed be presented by the classical MHCII pathway to activate CD4 T cells. A need for T cells for the production of carbohydrate allergen-specific IgE antibodies is also in line with the T cell dependency of the majority of high affinity antibody responses. During the course of the antibody response, both the effector functions of an antibody and the affinity for its cognate antigen are specifically adapted in so-called germinal center reactions, which are crucially depending on co-stimulatory signals as well as cytokines expressed by T helper cells. The affinity of the antibody is increased by somatic hypermutation of the variable regions and the subsequent selection of B cells with high affinity for their cognate antigen, a process called affinity maturation. The effector function of an antibody is mediated by its constant region, which determines the isotype of the antibody. All mature B cells initially express antibodies of IgM isotype, which can be modified by the process of class switch recombination initiated by co-stimulation as well as a defined cytokine milieu. In the case of high affinity IgE antibody responses, a possible sequential class switching of IgG1 to IgE has been proposed, in accordance with the limited and transient participation of IgE positive B cells in germinal center reactions and memory responses (85, 106–108).

## Glycolipid Allergen Sensing – NKT Cells Do the Job

While glycoproteins can be presented via the classical MHCII pathway, glycolipids are uniquely presented by the MHC Class I like molecule CD1 mainly expressed by B cells, dendritic cells and thymic T cells (109). Self and non-self lipids are in turn sensed by a separate T cell lineage, the NKT cells. NKT cells co-expressing natural killer (NK) cell markers and carry semi-invariant CD1-restricted αβTCRs on their surface (110). While humans express five different CD1 molecules which are organized into two classes based on sequence homologies (class I: CD1a, b, c; class II: CD1d), mice lack expression of the class I genes and thus solely express CD1d (111, 112). Upon ligand-CD1d recognition, NKT cells exhibit cytotoxicity mediated by Fas-Fas ligand interaction and rapidly secrete large amounts of cytokines including IFNγ, GM-CSF and the Type-2 cytokines IL-4, IL-5 and IL-13 (113, 114). The glycolipid alpha-galactosylceramide (α-GalCer), first identified in a marine sponge, is a well-known high affinity NKT antigen (115). Indeed, CD1d-α-GalCer tetramers stained up to 95% of human and mouse NKT cell clones (116). Additional CD1 ligands include microbial lipids such as glycosphingolipids and α-galactosyldiacylglycerols from the Gram-negative, LPS-negative bacteria *Sphingomonas* and *Borrelia burgdorferi*, respectively (117, 118), as well as self-lipids like the lysosomal glycosphingolipid isoglobotrihexosylceramide (iGb3) (119). Interestingly, while mice showed an increase in IFNγ as well as IL-4 and IL-10 after a single immunization with α-GalCer, repetitive immunisations resulted in a Th2 polarization of the immune response, with a dramatic reduction in IFNγ and an increase in IL-4 and IL-10 secretion, accompanied by an increase in serum IgE levels (120). Indeed, lipids from pollen, insects and food allergens have been described to play a role in allergic sensitization by direct recognition of these lipids via NKT cells. Agea et al. showed that phospholipids isolated from cypress pollen grains induced proliferation and secretion of both IFNγ and IL-4 in T cells isolated from allergic patients. Pollen grains directly interacted with dendritic cells and this interaction was blocked by anti-CD1d as well as anti-CD1a antibody treatment. Finally, NKT cell clones from cypress allergic individuals efficiently induced IgE production by autologous B cells *in vitro* and phospholipid-specific IgE antibodies could be detected in the serum of allergic but not control individuals (121). Another work by Abos-Gracia et al. showed that different lipids from olive pollen induce maturation of immature dendritic cells, accompanied by CD1d upregulation and IFNγ as well as IL-4 production, which in turn activated iNKT cells (122). By investigating PBMCs from food-allergic children, Jyonouchi et al. demonstrated that iNKT cells from allergic individuals produced higher amounts of the Th2 cytokines IL-4 and IL-13 in response to α-GalCer stimulation compared to iNKT cells from non-food allergic individuals. Stimulation of iNKT cells from milk-allergic individuals with milk sphingolipid- as well as α-GalCer-loaded CD1d tetramers specifically induced iNKT cell activation and proliferation, while egg ceramide-loaded CD1d tetramers did not. Interestingly, stimulation with milk sphingolipid- but not α-GalCer-CD1d tetramers resulted in clear IL-4 secretion by iNKT cells. When cultured in presence of milk sphingolipid, iNKT cells from allergic but not non-allergic children showed a clear Th2 response (123). Bourgeois et al. could show that phospholipase A2 from bee and wasp venom indirectly mediated a CD1a-restricted T cell response by cleaving cellular phospholipids which in turn served as neoantigens for CD1a-restricted T cell activation by antigen presenting cells (124). In a follow-up study, an increase in bee and wasp venom-reactive CD1a-restricted T cells with increased IFNγ, GM-CSF and IL-13 production in response to venom or venom-derived phospholipase was observed in venom-allergic individuals (125). Taken together, CD1-mediated presentation of lipids to NKT cells plays a crucial role in sensitization to allergens whereby the allergenic compound is either itself the lipid ligand presented on CD1 or is involved in lipid ligand processing.

## Sensing of Carbohydrate Allergens

In order to present allergens to T cells, APCs have to sense, take-up and process the allergen. Carbohydrate antigens are specifically recognized by a subset of glycan-binding proteins, containing carbohydrate recognition domains with specific binding grooves for certain self and non-self carbohydrate structures, the so-called lectins (126, 127). Among those, C-type lectin receptors (CLRs) essentially contribute to the pattern recognition ability of myeloid cells and mediate dendritic cell activation, antigen uptake and presentation to T cells. Interestingly, the allergy-promoting effects of some allergens such as the major peanut allergen Ara h 1 and the house dust mite allergen Der p 1 have been shown to clearly depend on their glycosylation and interaction with glycan-binding receptors on dendritic cells (128, 129). Shreffler et al. investigated the sensing, uptake and subsequent presentation of glycosylated vs. deglycosylated peanut allergen by dendritic cells and could show that recognition, uptake and subsequent activation of T cells was depending on the glycosylation status of peanut allergen and mediated by interaction of Ara h 1 with the C-type lectin receptor DC-SIGN on dendritic cells (128). Al-Ghouleh et al. investigated the capability of immature dendritic cells to take up Der p 1 and showed a carbohydrate-dependent uptake as well as interaction of Der p 1 with the CLR mannose receptor, which was increased when the allergen was hyperglycosylated but abolished after periodate treatment. Additionally, treatment of human lung epithelial cells with glycosylated but not periodate-treated Der p 1 resulted in increased secretion of the type-2 cytokine TSLP (129). Interestingly, beside their role in antigen recognition and uptake, CLRs can influence the polarization of T cell responses by inducing signaling cascades resulting in the expression of specific cytokines and chemokines as well as the modulation of signaling pathways induced by other (pattern recognition) receptors. Concerning DC-SIGN, it has been shown that distinct signaling circuits are induced depending on the recognized PAMP-associated carbohydrate. Thus, in response to fucose-expressing *Schistosoma mansoni* and *Heliobacter pylori*, DC-SIGN signaling via LSP1 induces a Th2-dominated response while inhibiting TLR4-induced pro-inflammatory cytokine responses such as IL-6 and IL-12. In contrast, sensing of mannose-expressing *Mycobacterium tuberculosis* or *HIV-1* resulted in an enhanced pro-inflammatory response (130, 131). Moreover, major peanut allergen Ara h 1-mediated T cell activation by dendritic cells resulted in a Th2-skewed response in a carbohydrate depending manner (128). Taken together, CLRs function as self and non-self carbohydrate-specific pattern recognition receptors involved in antigen uptake and presentation, activation of dendritic cells as well as the orchestration of individual immune responses, including the skewing of type 2 responses in the context of parasitic infections and allergies.

## Tolerance and Allergy to Carbohydrates – The Alpha-Gal Story

While eliciting Th2 immune responses in a few individuals, potential allergenic substances such as from pollen, food and mites are in general tolerated by the immune system. In the case of alpha-gal, clinical tolerance is the main finding in humans and exposure to alpha-gal from the environment is manifold through food and bacterial colonization of the surface organs, resulting in abundant levels of natural IgM and IgG antibodies directed against alpha-gal in human serum (38). These so-called natural alpha-gal specific antibodies are beneficial in the protection against pathogens carrying alpha-gal on their surface, suggesting that the frameshift mutation in the *Ggta1* gene and the associated loss in α-galactosyltransferase function in apes and humans provided an evolutionary advantage with respect to infections with alpha-gal carrying pathogens (132–134). Indeed, Yilmaz et al. showed that colonization of germfree mice with the alpha-gal-expressing *E.coli* strain O86 resulted in induction of alpha-gal-specific IgM and IgG antibodies which provided protection against malaria infection (47). However, in some individuals, repetitive tick bites can break this immune tolerance to alpha-gal by the induction of alpha-gal specific IgE (54, 67, 135). To date, the origin of the tick-transmitted alpha-gal carrying glycoproteins and glycolipids as well as the mechanisms inducing the IgE response remain elusive. Concerning the source of alpha-gal, tick saliva-derived proteins, mammalian proteins and glycolipids ingested by the tick during a previous blood meal as well as alpha-gal expressing bacteria, viruses or parasites potentially transmitted by the tick have been proposed (56, 57, 71, 136). Beside the transmission of alpha-gal carrying components to the host skin, tick bites induce a variety of host immune mechanisms which likely contribute to the induction of a type 2-dominated response in the case of alpha-gal allergy (137, 138). Tick saliva contains a variety of immunomodulatory molecules such as PGE_2_ and evasins which are involved in the suppression of pro-inflammatory immune responses, thereby favoring a Th2 polarization. Indeed, mice infested by ticks showed increased levels of both IL-10 and IL-4 which progressively increased with subsequent tick infestations, while IFNγ and IL-12 levels were reduced (139). Interestingly, PGE_2_ has been shown to be involved in the regulation of IgE class switching thereby enhancing IgE production by B cells (140). Thus, in the special case of alpha-gal and potentially other tick bite-mediated allergic responses, the tick bite itself may be critical for the induction of the type 2-dominated immune response resulting in the expression of allergen-specific IgE antibodies. After penetration of the skin barrier, carbohydrate allergens are likely phagocytosed by dendritic cells which in turn activate T cells in skin-draining lymph nodes. Although the exact mechanism of alpha-gal uptake by APCs remains unknown, Ristivojevic et al. showed that bovine serum albumin (BSA) uptake by human immature dendritic cells was significantly higher when the protein was decorated with alpha-gal while protein degradation was reduced, suggesting that the carbohydrate is specifically recognized by the dendritic cell inducing uptake and processing, possibly involving lectin-mediated recognition (141). As described above, T cell help was described to be crucial for the induction of alpha-gal-specific antibody responses (98). Thus, dendritic cells may migrate to the skin-draining lymph node to activate antigen-specific T cells which in turn activate their cognate B cells, resulting in production of allergy-eliciting alpha-gal-specific IgE antibodies. Class switching to IgE as well as differentiation of type 2 helper T cells crucially requires the presence of the cytokine IL-4 and thus an initial cellular source of this Th2-associated cytokine, potentially basophils. Indeed, basophils have been shown to be recruited to the site of tick bites in an IL-3 and CD4 T cell dependent manner (142) and basophil numbers were enriched in the skin of alpha-gal-allergic patients at the site of tick bites (143). Taken together, allergic responses to carbohydrate allergens likely rely, similar to protein allergens, on antigen presentation by APCs, either via the classical MHCII pathway or, in the case of glycolipids, via CD1 molecules to T cells, and the subsequent T cell-dependent induction of B cell responses. However, for a fundamental and more general understanding of the mechanisms and cell types involved in immunity to carbohydrate allergens including alpha-gal, further studies are urgently needed.

## Concluding Remarks

In this review, we described how the role of carbohydrates as mediators of allergic diseases changed over time, from crossreactive carbohydrate determinant to specific allergens eliciting anaphylactic responses. Especially the recent identification of alpha-gal as the allergen responsible for elicitation of allergic responses to red meat, innards and therapeutic monoclonal antibodies such as cetuximab changed researchers' view on carbohydrate allergens dramatically. However, the exact mechanisms involved in the break of tolerance as well as the elicitation of the allergic response to carbohydrate allergens are still elusive. Since the prevalence for allergic diseases is still increasing in developed and developing countries and specific therapies for allergic diseases are limited to the treatment of symptoms and the avoidance of the allergenic substance, new therapeutic tools are urgently needed. The development of such therapies in turn requires in-depth understanding of the immunological mechanisms behind recognition, presentation and initiation of the adaptive immune response to carbohydrate allergens, finally resulting in the production of allergy-eliciting IgE antibodies by allergen-specific B cells.

## Author Contributions

MH, FW, and TB took the lead in writing the manuscript. MH and FW designed the figures. CH, JF, and NH contributed relevant parts to the manuscript and provided critical feedback. All authors contributed to the article and approved the submitted version.

## Conflict of Interest

The authors declare that the research was conducted in the absence of any commercial or financial relationships that could be construed as a potential conflict of interest.
